# Spatial Vision and Visually Guided Behavior in Apidae

**DOI:** 10.3390/insects10120418

**Published:** 2019-11-22

**Authors:** Almut Kelber, Hema Somanathan

**Affiliations:** 1 Lund Vision Group, Department of Biology, Lund University, Sölvegatan 35, 22362 Lund, Sweden; 2 IISER TVM Centre for Research and Education in Ecology and Evolution (ICREEE), School of Biology, Indian Institute of Science Education and Research, Maruthamala PO, Vithura, Thiruvananthapuram, Kerala 695551, India

**Keywords:** honeybees, stingless bees, carpenter bees, social bees, solitary bees, foraging, mating, visual ecology

## Abstract

The family Apidae, which is amongst the largest bee families, are important pollinators globally and have been well studied for their visual adaptations and visually guided behaviors. This review is a synthesis of what is known about their eyes and visual capabilities. There are many species-specific differences, however, the relationship between body size, eye size, resolution, and sensitivity shows common patterns. Salient differences between castes and sexes are evident in important visually guided behaviors such as nest defense and mate search. We highlight that *Apis mellifera* and *Bombus terrestris* are popular bee models employed in the majority of studies that have contributed immensely to our understanding vision in bees. However, other species, specifically the tropical and many non-social Apidae, merit further investigation for a better understanding of the influence of ecological conditions on the evolution of bee vision.

## 1. Introduction

Apidae is the largest bee family (over 5900 species) and, besides Halictidae, has the largest number of social species including the approximately 500 species of stingless bees (subfamily Apinae; tribe Meliponini) as well as the 8 species of honeybees (genus *Apis*) and approximately 270 species of bumblebees (genus *Bombus*; tribe Bombini) [1,2]. Although social bees have attracted more attention than the even larger number of solitary bees, most studies on visual behavior and adaptations have focused on just two species, the European honeybee *Apis mellifera*, and the buff-tailed bumblebee *Bombus terrestris*. These two species, and more recently some species of stingless bees, have served as models for color vision (which we do not discuss here, for reviews see [3,4]), while other basic properties of their visual systems, such as spatial resolution, contrast sensitivity, and absolute sensitivity to light, have been studied to a lesser extent. Specifically, very few comparative studies have been performed. In this review, we aim to summarize the current knowledge on spatial vision for social Apidae, relate it to their flight activity and their visually guided behaviors, and compare it to what is known from some other bees.

Bees use a pair of large compound eyes and three small lens eyes, the ocelli, for visual tasks. The compound eyes of bees have a small dorsal region specialized for the perception of polarized light, called the dorsal rim. In this review, we do not discuss polarization vision, a topic excellently reviewed by others [5,6,7]. Behavioral tasks, such as finding flowers or mate detection, require high spatial resolution and are served by the main part of the compound eye [8], while others, likely served by ocelli or the dorsal rim region of the compound eyes, function with low spatial resolution. Low-resolution tasks include, for instance, phototaxis and the use of the polarization pattern of the sky as a compass for navigation (e.g., [7,9]). While most bees are active during daytime, few species have extended their activity into the night, which requires high absolute sensitivity of their eyes. Males may have different demands than females, and thus, dimorphic eyes occur amongst bees [10,11,12,13,14]. We will review properties of the eyes and vision first, and then discuss visually guided behaviors. 

## 2. Visual Fields, Sensitivity, and Resolution of the Apposition Compound Eyes of Bees

In the typical apposition eyes of bees, each ommatidium, with its own facet lens and 8 or 9 photoreceptors, is equivalent to a pixel in the image. Obviously, a larger eye can have more ommatidia, ommatidia with larger facets, or a combination of both, compared to a smaller eye [8]. Given the same visual field (but see [15]), this results in higher resolution, higher sensitivity, or a combination of both. Because each pixel is served by its own optical apparatus, sensitivity and resolution can vary widely within an eye, leading to acute and bright zones, the most extreme forms of which are found in the eyes of male bees [10,11,12,13,14]. When comparing different species, we mostly focus on the eye regions with highest resolution and sensitivity (Table 1).

### 2.1. Sensitivity

The sensitivity *S* of photoreceptors to broad-spectrum light is limited by four parameters: the area of the corneal facet lens (π/4 *D*)^2^, where *D* is the facet diameter, the focal length *f*, and the length *l* and distal diameter *d* of the photo-sensitive structure, the rhabdom, as summarized by Warrant and Nilsson [23]:

*S* = (π/4)2 *D*^2^ (*d*/*f*)^2^ (k *l*/(2.3 + k *l*) (1)

where k is the absorption coefficient of the rhabdom that is wavelength-dependent but—assuming a broad spectrum of light—can be taken as a constant (0.0067/μm, see [24]). Larger facets, and wider and longer rhabdoms, as well as a short focal length increase sensitivity. 

### 2.2. Resolution

Spatial resolution is limited by two parameters, one of them being the angle separating the optical axes of two ommatidia, the interommatidial angle. Baumgärtner [18] determined interommatidial angles in the honeybee eye using careful sections and measuring minimum angles between adjacent facet lenses of 0.9° in the vertical and 1.6° in the horizontal plane (but see [25]). Inter-facet angles have been used to approximate optical axes of ommatidia in recent micro-CT studies on bumblebees [26]. This works when the optical axes of ommatidia are normal to the corneal surface, which is the case in central eye regions, where resolution is highest. When optical axes are skewed, as in the periphery of bee eyes, inter-facet angles underestimate interommatidial angles, and with them the extension of the visual field (see [27] for a discussion of the problem and Figure 1D in [20] for an example). Using an optical method, interommatidial angles have been observed in female carpenter bees for facet rows of different orientations [21]. Minimal angles between horizontally running x facet rows, and the vertical to oblique y and z facet rows are, respectively, 0.90°, 1.82°, and 1.28° in *Xylocopa leucothorax*; 0.82°, 1.82°, and 1.28° in *X. tenuiscapa*; and 0.67°, 1.12°, and 0.81° in *X. tranquebarica*. 

The difference between horizontal and vertical interommatidial angles is similar amongst bees; it results from the oval shape of bee eyes and has been taken in consideration in models of bee vision (e.g., [28]). However, resolution is finally limited by the acceptance angle of receptors in each ommatidium, which can be approximated as:

Δϕ = *d*/*f* (2)

or, most accurately, be determined electrophysiologically. Rigosi et al. [17] measured mean acceptance angles of worker honeybee photoreceptors in the equatorial eye region as 2.2° in the horizontal and 2.3° in the vertical direction, and minimal acceptance angles down to 1.6°. If interommatidial angles are smaller than acceptance angles, the eye oversamples, which will not lead to higher resolution, but can potentially allow for higher sensitivity, if neural mechanisms of spatial pooling are in place [24,29,30]. 

### 2.3. Sensitivity, Resolution and Visual Field Size Depend on Body Size

**Figure 1:**
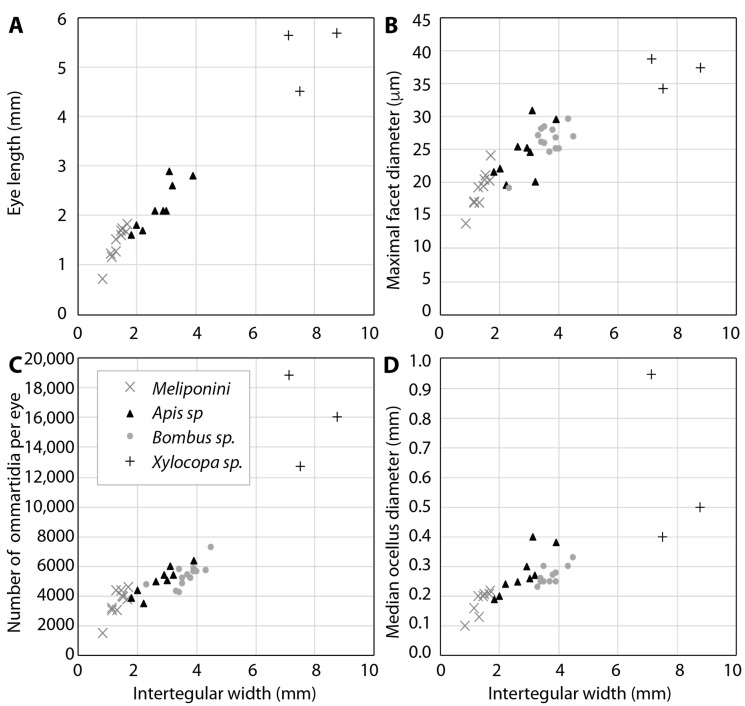
The dorso-ventral length of the compound eye (**A**), its maximal facet diameter (**B**), and number of ommatidia (**C**), as well as the diameter of the median ocellus (**D**) in differently sized female bees. Intertegular width is taken as an independent measure for body size. Data from [12,13,18,21,33]. Eye length was not measured in bumblebees. See [33] for statistical analyses of the relationship between body size and eye size.

**Figure 2:**
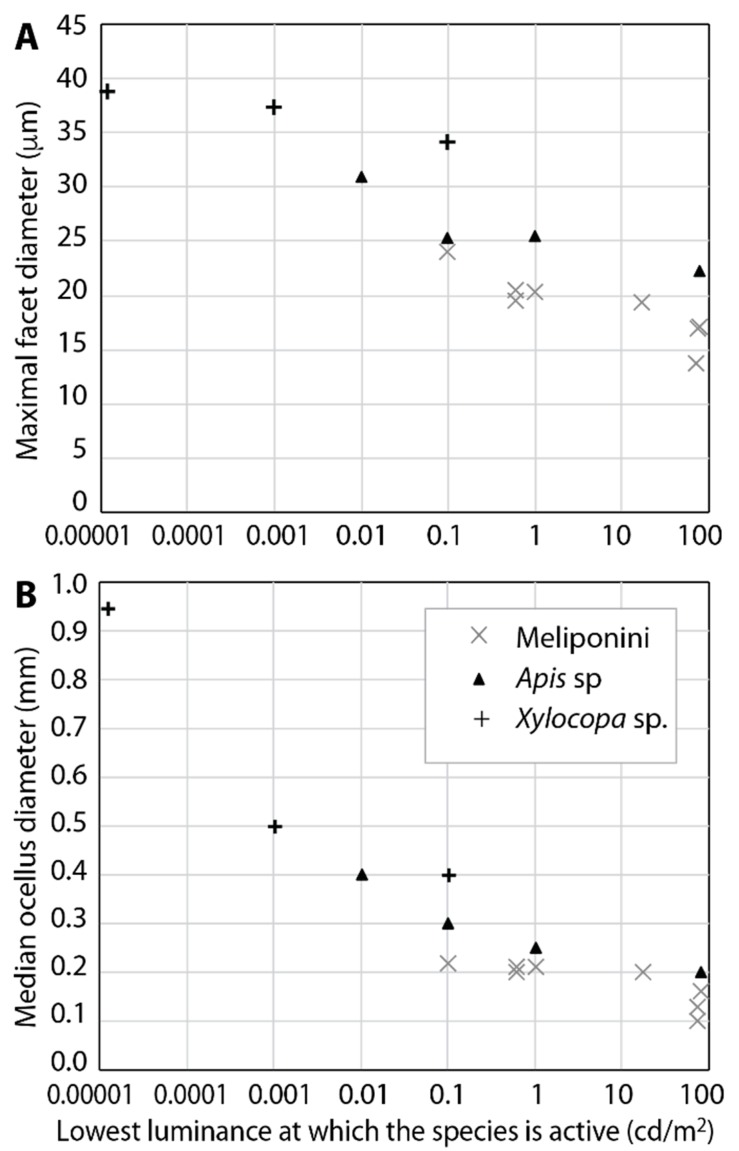
Ommatidial facet size (**A**) and median ocellus diameter (**B**) in bees flying at different light intensities: bee species with larger facets and larger ocelli fly at dimmer light intensities than species with smaller facets and smaller ocelli. Inset in (**B**) also applies to (**A**). Data from [18,21,33,102]. See [103] for a comparison of luminance and illuminance values.

In social bees, the eyes, and both the number of ommatidia in each eye and the ommatidial diameters, are larger in larger species (Figure 1A–C). Thus, both spatial resolution and sensitivity are higher in larger than in smaller species of bees, but crepuscular or nocturnal activity has an additional influence on facet diameters (Figure 2; see also [18,21,31,32,33]).

While honeybee and stingless bee workers of the same species are very similar in size and, thus, have similar eyes and visual capabilities, bumblebees [19] and many solitary bees (e.g., [34]) can have quite dramatic variations in body and eye sizes between individuals. According to Taylor et al. [26], eye size varies even more with body size, intra- than interspecifically, at least among bumblebees. This makes very small individuals of a bumblebee hive far less suited as foragers, compared to their larger nest mates. 

Besides resolution and sensitivity, the visual fields of bee eyes can also differ with body and eye size. Generally, the two compound eyes of bees have large visual fields, with a binocular region frontally and dorsally, and only a small blind region below and behind the animal. The European honeybee has a 50–60° (25–30° to the left and right of the frontal axis) binocular field in the eye region which looks forward in flight [35]. Large carpenter bees also have 40–50° binocular overlap at the eye horizon [21]. A detailed study of visual fields, however, has only been undertaken in bumblebees. Taylor et al. [26] show that larger individuals of *Bombus terrestris* may have larger visual fields and binocular overlap than smaller individuals, even though their method likely underestimates visual field size (see their Figure 1D). However, as this is the only study so far, more comparative work is required to better understand the behavioral and ecological relevance of differences in visual field sizes.

### 2.4. Sexual Dimorphism of Eyes

In social bees, in which workers, queens and males (drones) have rather different repertoires of visually guided behaviors, the eyes can differ distinctly. In females (queens and workers), resolution is highest in a fronto-horizontal region, in which they face flowers, the nest entrance, and other surfaces on which they land. The degree of sexual dimorphism depends partly on the mating system of a species. In bumblebee and carpenter bee species, which rely to a large degree on chemical cues, male and female eyes are more similar than in species that use visual mate detection [13,14,36]. 

Where sexual dimorphism is found, the eye region of highest spatial resolution in the drone eye, the acute zone, is often found in the dorsal part of the eye [10,11,12,13,14]. In *A. mellifera* drones, the upper third of the eye builds a large upwards-looking acute zone with larger facet diameters (30–40 μm), smaller inter-ommatidial angles (1–2°), and larger, and thus more sensitive, rhabdoms (2–3 μm^2^), than the remaining two-thirds of the eye (20–30 μm; 2–4°; 0.8–2 μm^2^) [10,11]. In *A. mellifera* drones, which have twice as many ommatidia in each eye than workers, the visual field is also expanded compared to females, because the two compound eyes meet at the dorsal border, and the lateral extension of each eye is larger (2.5 mm versus 1 mm). Enlarged eyes with dorsal acute zones are also found in the drones of other honeybee species, *Apis florea*, *A. dorsata*, *A. cerana*, and *A. andreniformis* (Table 2; and see [12]). The drones of the two open-nesting Asian species, *A. florea* and *A. dorsata*, have the most extreme adaptations for mate detection, with *A. dorsata* possessing enlarged ommatidia, and *A. florea* having smaller but more facets, an indication for spatial pooling. In carpenter bees, sexual dimorphism of eye size is correlated with mating strategy [36]. Males, which defend resources, tend to have dorsal acute zones with more and larger facets and smaller interommatidial angles than females, as described in *X. tenuiscapa* [14], while no obvious sexual dimorphism is seen in species with other mating strategies. A similar pattern can be seen in bumblebees: males of species, which adopt a perching strategy, for instance *B. confusus*, *B. melaleucus*, and *B. niveatus*, have larger eyes and facets than workers, while those using a patrolling strategy have similarly sized eyes ([13]; and see Table 2). Further studies of sexual dimorphism in bees will reveal which adaptations of the eyes are related to general properties of the habitat and which are related to the demands posed by sex-specific behaviors.

## 3. Ocelli and Their Function

Fronto-dorsally on the head, between the pair of large compound eyes, bees possess three small lens eyes, called ocelli [5,6,9]. Independent of their specific arrangement and position in different species, and partly as a result of different head angles during flight [6,15], all bee ocelli have similar, large visual fields covering the entire dorsal and frontal part of the world, even though hairs occlude part of the visual field in furry bees like *Apis* and *Bombus* [15]. Their large lenses (Figure 1D and Figure 2B) make ocelli more sensitive than the single ommatidia of the compound eyes. Consequently, honeybees [37] and bumblebees [38] with blinded ocelli start and stop foraging flights at about five times higher light intensities than bees with intact ocelli. That high absolute sensitivity of ocelli may be generally important is strengthened by the observation that nocturnal bee species have extremely large ocellar lenses, up to 1 mm in diameter (*Xylocopa tranquebarica*; [21]). 

Bee ocelli have irregularly shaped and often astigmatic lenses, whose focal plane usually lies behind the retina, resulting in relatively poor spatial resolution (e.g., [15,39]). The retina is bi-sectioned: the ventral retina is thin and views the—usually bright—sky above the bee, while the dorsal retina, which looks at the horizon of the flying bee, is usually thicker [6]. A prominent equatorial fovea-like indentation of the retinae is common, in which distal receptor endings are furthest away from the lens surface, and receptor densities are highest. The rhabdoms, each formed by rhabdomeres with short microvilli of two adjacent photoreceptors, build elongated, non-twisting plates, making the cells strongly polarization-sensitive [5,6,40]. 

Despite similarities across bees [6], there is also variation in structure, and likely function. Orchid bee ocelli are exceptional in having the dorsal retina within the focal zone of the lens, allowing for higher spatial resolution of the scene on front. In addition, microvilli within each ocellus are parallel, but oriented at 60° to those of the other two ocelli, making the ventral retinae a perfect polarization analyzer for the sky above the bee [41]. Honeybee ocelli have two types of photoreceptors, maximally sensitive to 500 nm and 360 nm [40]; the UV-green contrast is suitable to detect the sky-terrestrial boundary (e.g., [42]). The nocturnal halictid bee *Megalopta genalis* only has ocellar receptors with peak sensitivity at 500 nm and no polarization sensitivity, both likely adaptations increasing absolute sensitivity [5,43]. The nocturnal carpenter bee *X. tranquebarica* is the only described case in which the dorsal retina of the ocelli has evolved a tapetum (specifically, a tracheal tapetum) to enhance absolute sensitivity [21]. 

Ocelli can be sexually dimorphic (Table 2). In drones of most honeybee species, where they compete for space with the enlarged compound eyes, ocelli are slightly larger than in worker bees and have smaller visual fields, both increasing sensitivity [5]. The exception are giant honeybees *Apis dorsata*, in which the facultatively nocturnal workers have equally large ocelli as the drones [12]. In stingless bees, ocelli are understudied, but in one investigated species, males have significantly larger ocelli than both workers and queens ([10] and see Table 2). In *B. terrestris*, there are no signs of sexual dimorphism [15]. 

Bee ocelli likely serve several functions, including horizon detection and flight control by the dorsal retina [9] and evaluation of the polarized skylight by comparison of signals from the ventral retinae of all three ocelli ([38,40] but see [43]). These tasks are best served by highly sensitive receptors, large visual fields, and low spatial resolution. Probably related to their higher sensitivity compared to the compound eyes, ocelli allow bees to expand flight activity into dimmer light intensities [37,38]. Across bee species, ocellar diameters are well correlated with the light regime under which bees fly (Figure 2B; [33,44,45]). The fact that the ocellar interneurons of bees show a lower degree of convergence than those of some other insects [9], together with retinal adaptations [6], strongly indicates additional functions that require better spatial resolution. This is most obvious in the orchid bee [41], but more detailed studies of additional bee species are required for a broader understanding of ocellar function across bees. Specifically, comparisons of more bees foraging or performing other visually guided behaviours in different habitats and time windows may reveal further adaptations and the plasticity of ocellar function.

## 4. Visually Guided Behaviors in Bees

Almost all behaviors that bees perform outside the hive require some spatial resolution and are restricted by light intensity and the sensitivity of compound eyes and ocelli [31,32,33,43,44]. We limit this review to behavioral tasks guided by the main part of the eye and exclude the dorsal rim that serves the important task of extracting compass information from the polarization pattern of the sky, for which we refer the reader to the excellent review by Zeil et al. [7].

### 4.1. Nest Defense 

Social Apidae are known to guard their nest entrance from intruders. In the stingless bee *Tetragonisca angustula*, guard bees hover in front of the nest in stable positions to protect the flight path from intruders. Positioning is under visual control, thus, when presented with an expanding pattern of stripes or a rotating spiral at the nest front, bees flew away from the nest, whereas, with a contracting pattern, they flew closer to it [45,46]. Identification of intruders, however, is also chemically mediated [47]. The giant honeybee *Apis dorsata*, which inhabits large open combs in high trees, rocks, and buildings, also uses visual cues in nest defense. Guard bees sitting on the comb approach and attack objects (potential intruders) that move towards the hive. In experiments with paper disks, Koeniger et al. [48] showed that the guard bees react much more strongly to objects moving upwards than to objects moving downwards, likely because the latter are perceived as harmless objects, such as falling leaves.

### 4.2. Use of Landmarks for Navigation and Homing

When a bee leaves the hive for the first time, she performs a learning flight, which allows her to find her way back to the nest (e.g., [49,50,51,52,53,54,55,56]). Even during later foraging periods, she updates this information whenever local features change in the vicinity of the nest. These flights have been synonymously referred to as orientation flights, learning flights, or turn back and look behavior (e.g., [49,50,51,52,53]. This behavior, which has been largely documented in *A. mellifera* [50,51,52,53] and *B. terrestris* [49,54,56,57], helps a forager learn features around the nest or a feeding location when viewed from different positions. Orientation flights are typically quite restricted to a narrow range around the goal point and as a bee gains experience, she flies faster during these flights; honeybee workers extend the range of these flights to cover increasing distances over time [53]. 

While performing these flights, a bee typically exits the nest or feeding location, turns around to face it while backing away in a series of successive arcs that are approximately centered on the point of interest i.e., the nest or feeding location. With experience, the bee will fly out in a straight line and will perform orientation flights only when local landmarks around the point of interest are disturbed. The principal function of these flights is to learn to recognize salient locations; thus, studies have compared outbound and return flights for correspondence in ground-nesting bees and in bumblebees (e.g., [54,55,56]). In the bumblebee *Bombus terrestris*, loops are components of learning flights that are more common when the bee is outbound than during return flights. On the other hand, zigzag flight paths are more prominent during return flights. A common feature to both loops and zigzags is that bees often face the nest such that it is held within the fronto-lateral visual field [56]. These findings suggest that bees can store and recall nest-centered views during learning and return flights. Interestingly, male bumblebees were found to perform learning flights only when leaving a food source and not the natal nest, suggesting inter-sexual differences in the value of the nest to males and females [57]. 

### 4.3. Foraging: Flower Detection

The best-studied function of the visual system of bees is the spatial resolution for circular or flower-shaped single targets. In the context of flower visits, experiments have asked from which distance—or at which visual angle—bees can detect and discriminate such targets. While the focus often was on color discrimination [3,4], a lot has been learned about spatial resolution using behavioral methods (Table 3; e.g., [19,28,58,59,60,61]). As eye size, ommatidial diameter and the number of ommatidia in each eye are correlated with body size (see above and Figure 1A–C), the small stingless bee *Tetragonula carbonaria* can detect flowers from the background only when they have almost a 10° visual extension, meaning they can detect a flower of diameter 1 cm at a distance of 6 cm only [20]. A European honeybee can detect the same flower when it fills 3° or 5° of their visual field, thus from a distance of 12 to 18 cm (e.g., [28,59,60]), and a medium-sized bumblebee can detect this flower from a distance of 30 cm, when it subtends 1.8° (see Table 3; [62] but see [19] for the effect of body and eye size variation). Spatial resolution of the large carpenter bees is likely even higher but has not been determined behaviorally [63]. 

Spatial contrast sensitivity, which relates contrast sensitivity and spatial resolution, has been behaviorally estimated only rarely in bees, by finding contrast sensitivity thresholds for gratings of different spatial frequencies [58,66].

### 4.4. Flight Ranges and Flight Control 

It can be expected that in bees using visual cues for navigation, flight range may at least partially depend on visual abilities. Since flying bees can seldom be tracked in nature (but see [69,70]), foraging distances have been estimated using release-homing experiments with marked individuals or by training bees to feeders at set distances and recording the distance at which bees cease to visit the feeder (Table 4). Four species of neotropical stingless bees were able to find artificial feeders placed between 320 and 680 m from the hive and also to return to the nests when released 600–800 m away from nests [71]. Alternatively, in honeybees, the distance travelled can be inferred from waggle dances [72]. When more than one method was deployed for a bee species, the estimated foraging distances differed somewhat (Table 4). Foraging distances are affected by landscape structure, as has been shown by Steffan-Dewenter and Kuhn [73], who compared European honeybees in grasslands, forests, arable land, and built up areas in Germany. The mean foraging distance estimated from waggle dances was 1.5 km [73]. 

Meta-analyses suggest a positive non-linear correlation between body size and foraging distances [83,85]. Nevertheless, apart from *Apis mellifera*, foraging distances are not well known in most Apidae species. The few studies that have examined Asian honeybee species, for instance, suggest considerable within- and between- site variation in foraging distances [72,86]. 

Bees, like many flying animals, use optic flow to measure distance flown on their way to and from food sources [87]. Optic flow is measured as the amount of image motion in the lateral visual field of the bee’s eye while flying towards a goal. Environments with many contrasting structures generate greater optic flow than those with fewer visual contrasts, suggesting that the bee’s perception of the distance flown varies in relation to the landscape [87,88]. This was investigated in honeybees and bumblebees flying through experimental tunnels, in which patterns on the walls of provided optic cues (e.g., [68,89,90]. Bees estimate flight distance using only the green receptor contrast of the patterns, independent of chromatic contrast and brightness contrast [91], and because translational optic flow depends on distance, the estimate depends on the distance of bees to the contrasting patterns (e.g., [92]). Bees also use optic flow to control flight speed and flight height [93], and to avoid flying too close to obstacles, by balancing the optic flow on both sides (e.g., [64,89]). In tunnel experiments, this tendency is obvious as the ‘centering response’: bees fly in the middle of the tunnel with symmetric optic flow, but close to one of the walls if it provides no optic flow cues [64,68]. Tunnel experiments also indicated that the stingless bee *Melipona panamanica* uses optic flow to gauge distance as well as the height of food sources [94]. The nocturnal sweat bee, *Megalopta genalis*, also uses optic flow in experimental tunnels [95]. This nocturnal bee flew five times slower than day-active bumblebees, and increased groundspeed when motion cues were reduced in the tunnel, though it did not their affect centering response. 

### 4.5. Male Mating Flights: Detection of Females

Besides homing (at least in some species), and finding and returning to flowers, the only important—and most demanding—visual task that a male bee has to perform is finding a mate. Males seek out mates at sites where females emerge, at food resources and at non-resource locations such as landmarks and flyways [96]. Studies on mating behavior of bees are sparse, however, whether or not this task involves visual cues can often be guessed from sexually dimorphic eyes (e.g., [36]). 

The high resolution and contrast sensitivity of receptors in the dorsal acute zone of drone honeybees *Apis mellifera carnica* allows a queen in flight to be detected against the sky even when she darkens just a small part of the visual field of a single ommatidium by about 6% [97]. Honeybee drones use the upper frontal part of the visual field to chase queens and they fixate the queen with the lower part of their frontal visual field where the ommatidial diameter is maximum [98]. 

More generally, the male eyes suggest an influence of mating flight timings, body size limitations, and the relative roles of vision and olfaction in mating behavior of various bee species. Drones of different honeybee species perform mating flights at different, barely overlapping times of the day, with the large *A. dorsata* being the only species using the dimmer time window after sunset (18:30–19:00 h in Thailand, during February) [99].

Mating behavior also varied in three sympatric species of carpenter bees that are diurnal (*Xylocopa tenuiscapa* and *X. leucothorax*) or nocturnal (*X. tranquebarica*) [14]. *X. tenuiscapa* perched outside nests early in the season and patrolled or perched along flyways and close to flowering plants later in the season. *X. leucothorax* patrolled along flyways, while the nocturnal *X. tranquebarica* patrolled close to flowering bushes. Perching *X. tenuiscapa* males can detect females flying at a distance of 20 m, which darkens the visual field of a single ommatidium by only 2%, suggesting that these carpenter bee males have similar or higher contrast sensitivity than honeybee drones [14,97]. 

## 5. Light Intensity and the Sensitivity of the Eyes

Most species of bees are diurnal, limited to the day for foraging and other activities performed outside the nest. This limitation could be due to lower temperatures or low light intensities at night or both. While temperature differences between day and night can be marked in temperate and arctic climates and in dry habitats, they are much smaller and less likely to limit bee flight in humid and tropical areas [90]. Thus, bees are generally more limited by light intensities than low temperatures [31,32,33].

Therefore, it is not surprising that the lens diameter of both types of bee eyes—the facet lenses of the compound eyes, and the ocellar lenses—and thus their sensitivity, dictate the lowest light intensity in which a species can be active ([31,33,44] and see Figure 2). In a recent study on the stingless bee *Scaptotrigona depilis* [100], for instance, the relationship between flight activity and light intensity was highly significant, while the relationship with temperature was not. Similarly, Heard and Hendrikz [101] reported that, in the Australian species *Tetragonula carbonaria*, commencement of flight activity is determined by temperature during the colder months of the year, but by light intensity during the warmer months, and that light intensity always determined the end of flight activity in the afternoon. 

Streinzer et al. [33] proved this to be true for a larger sample of stingless bee species, and Somanathan et al. [18] showed that among the four studied species of honeybees, *Apis dorsata* has the most sensitive eyes. *A. dorsata* is facultatively nocturnal—by contrast to the closely related species *A. laboriosa* [104]—and can forage during full moon nights [102], and is the only species in which drones perform mating flights during dusk [99]. However, the most intriguing example of nocturnality known among Apidae studied so far is the carpenter bee *X. tranquebarica*, which is able to forage and return to the nest on new moon nights [21,105], and can even use color under these conditions [106]. Studies on more species may reveal additional species which can change their activity window to the dimmer times of the day, likely escaping competition and improving foraging efficiency. 

A move to the nocturnal niche is possible for smaller species of bees (see, for instance [30,31,44] for examples of crepuscular or nocturnal bees and wasps with relatively small body size). This is mostly due to adaptations in the neural processing of visual information, which allow for spatial and temporal pooling of visual signals, both of which increase sensitivity [22,24,29,30]. 

## 6. Concluding Remarks

Social bees of the family Apidae, the most important pollinators of wild flowers and crops, rely on vision for many crucial behavioral tasks. A considerable body of research on the two European temperate species, *Apis mellifera* and *Bombus terrestris,* has accumulated over the years; the few studies on other bumblebees—including *B. impatiens*—and the large diversity of tropical Apidae indicate interesting differences, even though their compound eyes and ocelli all follow the same building plan. Most importantly, eye size, and with it, both absolute sensitivity and spatial resolution, scale with body size. The investment in eyes and ocelli, and subtler differences in the compromise between high spatial resolution and high absolute sensitivity to light, differ between both sexes and between bees active in different light levels. We expect that more adaptations, as well as differences in visual field size, will be found once more species are investigated. Such studies may reveal interesting and hitherto unreported adaptations to behavioral tasks and habitats, such as forests or open meadows. From the perspective of pollination biology, the relationship between body and eye size, and the foraging range of different bee species, is another topic that requires further investigation, as it may facilitate optimal pollinator augmentation.

We have focused on social Apidae, partly because their eyes and vision have been studied most thoroughly. We conclude that far too little is known about the eyes and visual abilities of the majority of bee species and suggest that comparative studies, both between temperate and tropical Apidae, and between solitary and social species, are required for a deeper understanding of how different ecological conditions have shaped the evolution of bee vision, behavior, and pollination services.

## Figures and Tables

**Table 1:** Ommatidial values in the eye regions of highest spatial resolution and resulting optical sensitivity of female bees. (a) anatomical estimation, (e) electrophysiological measure, (c) corneal angles, (po) pseudopupil othrodromic illumination, (pa) pseudopupil antidromic illumination, (h) horizontal, (v) vertical (or close to vertical, taking average of y and z rows). For additional information and methods descriptions please see the text and references.

Species	Intertegular Width (mm)	Interommatial Angle	Acceptance Angle (°)	Sensitivity S (m^2^ sr)	References
*Apis mellifera*	3.2	0.9° v/1.6° h (c)	1.7 (a), 1.6 (e)	0.11	[16,17,18]
*Apis cerana*	3.0		1.2 (a)	0.07	[18]
*Apis florea*	2.2		1.1 (a)	0.03	[18]
*Apis dorsata*	3.9		1.8 (a)	0.21	[18]
*Bombus terrestris*	small 3.0 large 4.2	1.2 v/2.9 h (pa) 0.9 v/2.1 h (pa)			[19]
*Tetragonula carbonaria*		1.3 v/2.3 h (pa)			[20]
*Xylocopa leucothorax*	7.5	0.9 v/1.5 h (po)	0.8 (a)	0.1	[21]
*Xylocopa tenuiscapa*	8.8	0.8 v/1.5 h (po)	1.1 (a)	0.3	[21]
*Xylocopa tranquebarica*	7.1	0.7 v/1.0 h (po)	2.7 (a)	2.7	[21]
*Megalopta genalis*	2.8	1.4 (po)	5.6	2.7	[22]

**Table 2:** Sexually dimorphic eyes and ocelli are common amongst Apidae. For additional details please see the references.

Species	Sex/Caste	Maximal Facet Diameter (m)	Number of Facets/Eye	Minimal Interommatial Angle	Median Ocellus Diameter (mm)	References
*Apis mellifera*	queen	26.1	4460		0.30	[10,11,12,16,25]
	worker	25.2	5375	1.6	0.28	
	drone	40.1	9993	1.0	0.34	
*Apis cerana*	queen	25.9	3582		0.27	[12]
	worker	25.4	4921		0.25	
	drone	35.8	7994		0.30	
*Apis florea*	queen	24.9	4036		0.27	[12]
	worker	22.1	4394		0.20	
	drone	38.0	9434		0.32	
*Apis dorsata*	queen	34.7	4479		0.38	[12]
	worker	30.8	5974		0.40	
	drone	46.3	8383		0.40	
*Bombus pratorum*	queen	30.1	5805		0.30	[13]
	worker	27.1	4301		0.23	
	male	28.5	4492		0.25	
*Bombus terrestris*	queen	29.3	7691		0.38	[13]
	worker	25.1	5656		0.28	
	male	27.4	5624		0.31	
*Bombus melaleucus*	queen	36.9	8528		0.39	[13]
	worker	29.5	5659		0.30	
	male	39.3	8299		0.36	
*Bombus niveatus*	queen	28.8	8617		0.42	[13]
	worker	26.8	7230		0.33	
	male	36.4	8051		0.34	
*Bombus wurflenii*	queen	32.6	6960		0.34	[13]
	worker	27.9	5213		0.27	
	male	28.6	5604		0.30	
*Bombus lapidarius*	queen	29.9	6765		0.38	[13]
	worker	25.9	4800		0.30	
	male	29.3	5214		0.30	
*Bombus hortorum*	queen	30.2	7010		0.31	[13]
	worker	28.4	5170		0.25	
	male	28.0	5232		0.25	
*Bombus pascuorum*	queen	32.2	6426		0.33	[13]
	worker	28.0	5803		0.25	
	male	29.1	5666		0.27	
*Bombus soroeensis*	queen	28.8	6042		0.31	[13]
	worker	26.0	4250		0.24	
	male	27.8	4968		0.26	
*Bombus confusus*	queen	29.7	7569		0.35	[13]
	worker	26.7	5870		0.25	
	male	39.2	7821		0.33	
*Bombus mendax*	queen	28.1	6868		0.35	[13]
	worker	24.5	5375		0.25	
	male	34.0	7032		0.28	
*Scaptotrigona postica*	queen	19	3800		0.24	[10]
	worker	21	3900		0.22	
	drone	21	4500		0.29	
	queen	19	3500		0.24	
*Xylocopa tenuiscapa*	female	37.3	15,994	1.0	0.50	[14,21]
	male	48.0	15,751	0.7	0.60	
*Xylocopa leucothorax*	female	34.2	12,716		0.40	[14,21]
	male	35.0	11,331		0.40	
*Xylocopa tranquebarica*	female	38.7	18,804		0.95	[14,21]
	male	40.0	15,511		0.90	

**Table 3:** Behaviorally determined thresholds of spatial resolution in bees. (a) values for point objects are calculated taking the visual angle obtained by the object as half the resolvable wavelength, allowing for direct comparison with the other data sets. Thus, bees can detect objects subtending half the diameter indicated here.

Species	Stimulus	Behavioral Response Tested	Minimum Spatial Wavelength (deg)	Minimum Spatial Frequency (Cycle deg^−1^)	Reference
*Apis cerana*	Sine wave stationary gratings	Object discrimination	2.8–3.8	0.26–0.36	[61]
	Sine wave gratings	Flight control, centering response	1.2–8.3	0.12–0.8	[64]
*Apis mellifera*	Gratings, bright light	Object discrimination	4	0.25	[24]
	Gratings, dim light	Object discrimination	8.3	0.12	[24]
	Square object	Object discrimination	5.7 (a)	0.18	[16]
	Square wave gratings	Optomotor response, walking bees	2.1	0.48	[65]
	Square wave grating	Object discrimination	4	0.25	[58]
	Point object	Object detection	6–11 (a)	0.09–0.17	[28,59,60,62]
	Sine wave gratings	Flight control, centering response	5.5–8	0.12–0.18	[64]
*Bombus terrestris*	Point object	Object discrimination	3.6–14 (a)	0.27–0.07	[19,62]
	Sine wave gratings	Object discrimination	4.8	0.21	[66]
	Sine wave gratings	Flight control, centering response	4.8	0.21	[64]
*Bombus impatiens*	Sine wave gratings	Object discrimination	2.8–2.9	0.35–0.36	[67]
	Sine wave gratings	Flight control, centering response	7.1	0.14	[68]
*Tetragonula carbonaria*	Point object	Object detection	18.8 (a)	0.053	[20]

**Table 4:** Foraging distances estimated for different Apidae species. For more information on methods, please see the references.

Species	Method Used to Infer Foraging Ranges	Average Foraging Distance (m)	Maximal Distance Estimate (m)	Reference
**Honeybees**				
*Apis florea*	Mark-recapture	150–250	750	[74]
	Dances	268	>800	[72]
*Apis cerana*	Dances	195	1200	[72]
	Feeder	650	1423	[75]
*Apis dorsata*	Dances	863	1000	[72]
*Apis mellifera*	Dances		10,000	[72]
	Dances	2300	approx. 8000	[76]
	Dances	1570	10,000	[73]
	Dances	5500	>10,000	[77]
**Bumblebees**				
*Bombus muscorum*	Mark-recapture	55	125	[78]
*Bombus lapidarius*	Mark-recapture	260	1500	[78]
*Bombus terrestris*	Mark-recapture	663	1750	[78]
	Mark-recapture		1500	[79]
	Harmonic radar		630	[80]
**Carpenter bees**				
*Xylocopa flavorufa*	Radio-transmitter		6040	[81]
*Xylocopa violacea*	Mark-recapture		1200	[82]
**Stingless bees**				
*Plebeia droryana*			540	[83]
*Melipona compressipes*			2470	[83]
*Trigona spinipes*			840	[83]
*Melipona quadrifasciata*			2000	[83]
*Melipona fasciata*	Release		2085	[84]
*Trigona capitata*	Release		1547	[84]
*Trigona corvina*	release/feeder		590/320	[71]
*Tetragonisca angustula*	release/feeder		662/680	[71]
*Nanotrigona testaceicornis*	release/feeder		484/120	[71]
*Paramona cff cupira*	release/feeder		622/520	[71]
